# Disinfection of Maternal Environments Is Associated with Piglet Microbiome Composition from Birth to Weaning

**DOI:** 10.1128/mSphere.00663-21

**Published:** 2021-09-08

**Authors:** Kayla Law, Brigit Lozinski, Ivanellis Torres, Samuel Davison, Adrienne Hilbrands, Emma Nelson, Jaime Parra-Suescun, Lee Johnston, Andres Gomez

**Affiliations:** a Department of Animal Science, University of Minnesota, St. Paul, Minnesota, USA; b University of Minnesota, West Central Research and Outreach Center, Morris, Minnesota, USA; c National University of Colombia, Medellin, Colombia; University of Michigan—Ann Arbor

**Keywords:** disinfection, environment, gut, maternal, microbiome, piglet, programming, swine

## Abstract

Maternal factors predetermine offspring development and health, including the establishment of offsprings’ first microbiomes. Research in swine has shown that early microbial exposures impact microbiome colonization in piglets, but this phenomenon has never been tested in the context of delivery room disinfection. Thus, we exposed gestating sows to two delivery environments (*n* = 3/environment): stalls cleaned with a broad-spectrum disinfectant (disinfected environment [D]) or stalls cleaned only with hot-water power washing (nondisinfected environment [Nde]), 3 days prior to farrowing. Microbiomes of sows and farrowed piglets (*n* = 27/environment) were profiled at 4 different time points from birth to weaning via 16S rRNA sequencing. The results show that although vaginal, milk, skin, and gut microbiomes in mothers were minimally affected, sanitation of farrowing stalls impacted piglet microbiome colonization. These effects were mainly characterized by lower bacterial diversity in the gut and nasal cavity, specifically in D piglets at birth, and by distinct taxonomic compositions from birth to weaning depending on the farrowing environment. For instance, environmental bacteria greatly influenced microbiome colonization in Nde piglets, which also harbored significantly higher abundances of gut *Lactobacillus* and nasal *Enhydrobacter* at several time points through weaning. Different sanitation strategies at birth also resulted in distinct microbiome assembly patterns, with lower microbial exposures in D piglets being associated with limited interactions between bacterial taxa. However, increasing microbial exposures at birth through the lack of disinfection were also associated with lower piglet weight, highlighting the importance of understanding the trade-offs among optimal microbiome development, health, and growth performance in swine production systems.

**IMPORTANCE** We show that levels of disinfection in farrowing facilities can impact early microbial exposures and colonization by pioneer microbes in piglets. Although previous research has shown a similar effect by raising pigs outdoors or by exposing them to soil, these practices are unattainable in most swine production systems in the United States due to biosecurity practices. Thus, our results underscore the importance of evaluating different disinfection practices in swine production to safely reduce pathogenic risks without limiting early microbial exposures. Allowing early exposure to both beneficial and pathogenic microbes may positively impact immune responses, reduce the stressors of weaning, and potentially reduce the need for dietary antimicrobials. However, the benefits of modified early microbial exposures need to be accomplished along with acceptable growth performance. Thus, our results also provide clues for understanding how disinfection practices in farrowing rooms may impact early microbiome development and assembly.

## INTRODUCTION

Maternal programming refers to the broad set of maternal characteristics and factors that can effectively predetermine and preprogram offspring health and development (1). This concept has been well studied in humans in the context of the differential effects of delivery modes (2), feeding methods after birth (3), and delivery environment (4) on the compositions of infant gut microbiomes. Maternal diets also influence offspring microbiome composition (5), with high fat intake during pregnancy being associated with offspring diabetes (6). Although the effects of perinatal environmental conditions on offspring gut microbiome composition may be limited to only the first year of life (7), long-term impacts on health have been proposed, with birth by Cesarean section being associated with increased risks of type 1 diabetes (8) and allergic disorders (9).

The investigation of factors that shape the seeding and composition of piglet microbiomes at birth represents a new approach to researching potential methods for improving swine health and productive performance. Pork production in the United States generates over $20 billion of economic activity annually (10). Continuous improvement of pork production systems requires evaluation of diverse management methods to ensure optimal pig health and performance. For instance, the effects of maternal nutrition and milk quality on piglet microbiomes and health have been well documented. Notably, maternal yeast supplementation is associated with elevated milk immunoglobulin content (11), a factor linked to piglet health and growth performance (12), as well as beneficial gut microbiome modifications for piglets (13). Likewise, a few reports have indicated that manipulation of the environment and microbial exposures during farrowing and rearing periods impacts the seeding and development of microbiomes and the immune performance of piglets. Environmental and microbial exposures through agricultural soil exposure (14), outdoor versus indoor rearing (15, 16), and access to maternal feces (17) demonstrate that a wide variety of microbial exposures early in life, including potential pathogens, increases immunocompetence and immune responses throughout the offspring’s lifetime (18). However, due to biosecurity concerns and costs, many of these strategies, such as outdoor rearing or exposure to soil during farrowing, are unattainable in commercial, large-scale confinement swine production systems.

Here, we investigate strategies to modulate early seeding of the piglet microbiome by manipulation of sanitation and disinfection in farrowing rooms. Farrowing room disinfection with broad-range microbicides, a standard operating procedure in swine production systems, restricts the levels of infectious microorganisms in the environment and reduces disease risks for sows and newborn piglets (19). However, we hypothesized that disinfection of the birth environment could also restrict broad microbial exposures, impacting the seeding and development of the piglets’ microbiome and, potentially, their physiological performance. To test this hypothesis, we characterized microbiome compositions of sows and piglets in farrowing stalls sanitized with hot-water power washing plus disinfection with a commonly used, broad-spectrum microbicidal and compared their microbiome profiles with those of sows and piglets in farrowing stalls that were sanitized with only hot-water power washing. The effects of these two sanitation strategies on microbiome composition were tested in the environment and in sows and piglets at different anatomical sites and at sequential time points. The results demonstrate a significant impact of perinatal disinfection on the composition and diversity of the nasal and gut microbiomes of piglets from birth to weaning as well as a potential effect on growth performance.

## RESULTS

**FIG 1:**
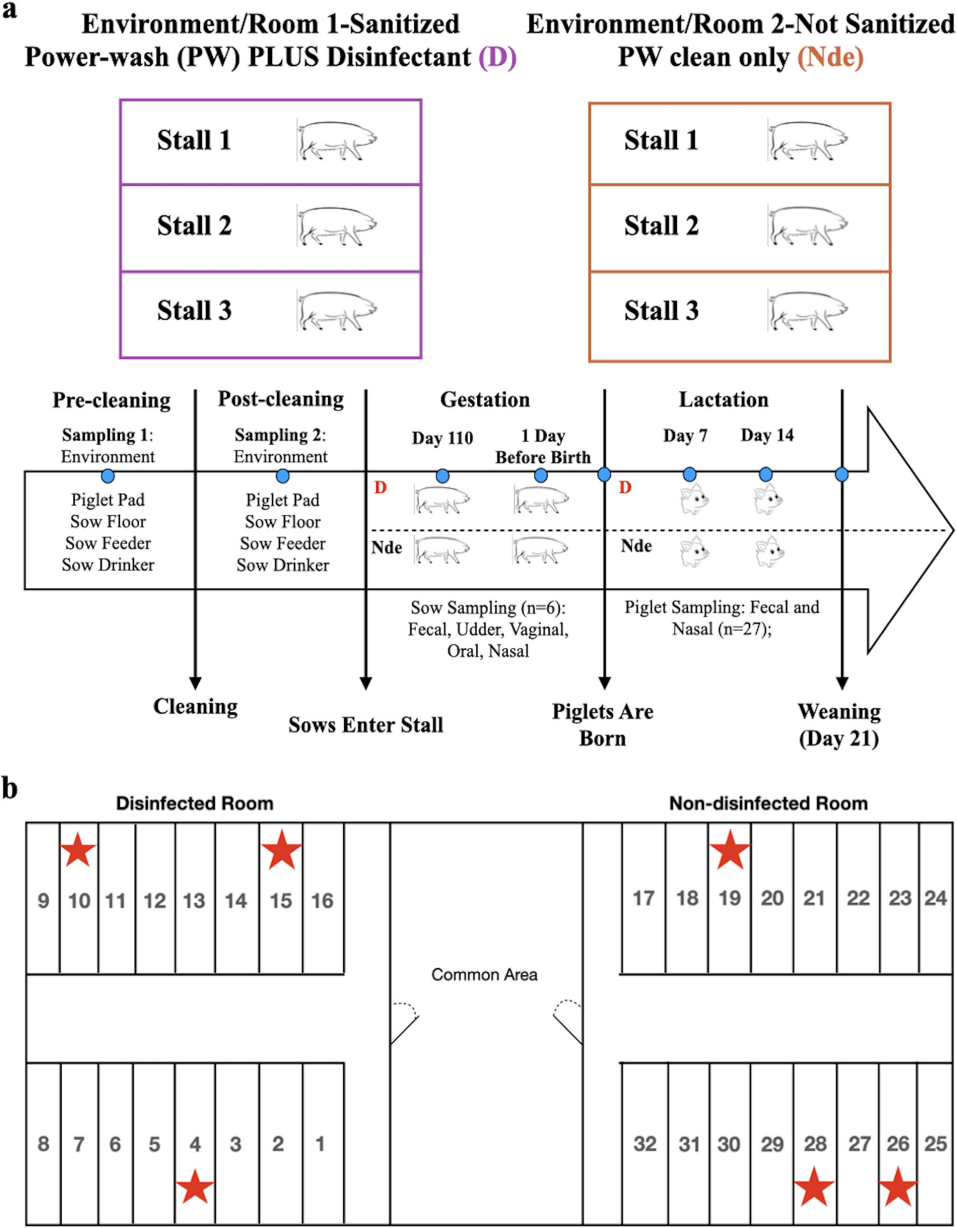
(a) Methodology and sample collection timeline for sows and piglets in disinfected (D) and nondisinfected (Nde) environments. (b) Diagram of the farrowing barn setup showing the separation of treatment groups into different rooms, with red stars denoting stalls randomly selected for the study.

Landrace × Yorkshire crossbred sows (*n* = 6) were separated into two treatment groups: one composed of farrowing stalls sanitized with hot-water power washing plus a commonly used disinfectant (disinfected environment [D]) (*n* = 3) and one composed of farrowing stalls where sanitation consisted of only hot-water power washing (nondisinfected environment [Nde]) (*n* = 3). Samples were collected from environmental surfaces within farrowing stalls, various sow body sites, and piglets at various time points (Fig. 1a). Farrowing stalls belonging to different treatment groups were located in separate rooms with strict biosecurity lines enforced to prevent cross-contamination between treatments (Fig. 1b). Specifically, swab samples were collected from four stall surfaces: farrowing stall floors under sows, heating pad surfaces in piglet creep areas, drinkers, and feeders. Samples were collected from one dirty farrowing stall before cleaning, one farrowing stall that was washed and disinfected, and one farrowing stall cleaned through hot-water power washing only to measure the effect of disinfection on the microbiome of the environment (*n* = 12). Vaginal, rectal, oral, and udder surface samples were collected from each of the six sows at day 110 of gestation upon stall entry and the day before farrowing. Milk was collected from sows at day 7 postpartum (*n* = 6). Swabs were collected from the interior of the rectal and nasal cavities of 9 piglets per sow (*n* = 54; 27 per farrowing environment) at day 0 (within 24 h of birth) and days 7, 14, and 21 postpartum, resulting in 108 total piglet samples collected from each farrowing environment (*n* = 216 total samples). DNA was extracted from swabs, and the V4 variable region of the 16S rRNA bacterial gene was sequenced on the MiSeq platform.

### Sanitation alters environmental microbiomes in farrowing stalls but not those of sows.

The sanitation method, either hot-water power washing or hot-water power washing plus disinfection, altered the bacterial biomass in farrowing stalls, as revealed by the tendency toward lower quantitative PCR (qPCR) copy numbers of the 16S rRNA gene in the disinfected environmental samples (see Fig. S1a in the supplemental material). Analyses of the number of amplicon sequence variants (ASVs) present in the environment (alpha diversity, adjusted by sequencing depth) also showed a tendency toward lower environmental bacterial richness after cleaning, whether through hot-water power washing with or without disinfection (Fig. S1b). The compositions of the environmental bacterial communities differed among dirty, disinfected, and nondisinfected environments postcleaning (Fig. S1c), with disinfection status, followed by surface type, driving the ordinations of community compositions. Because samples from the precleaning and nondisinfected postcleaning farrowing environments appeared to overlap in the ordination, constrained by the substantially different community compositions from disinfected surfaces (Fig. S1c), these samples were then presented in a separate ordination, demonstrating alterations in community compositions postcleaning, even without the use of a disinfectant. No effects of farrowing stall disinfection were observed in gut, vaginal, skin, milk, or oral samples of sows. This observation could be due to the small sample sizes per environment but also could be due to higher interindividual variation in samples from sows in disinfected stalls than in those in nondisinfected stalls (Fig. S1d to j).

10.1128/mSphere.00663-21.2 FIG S1 (a) Environmental samples postcleaning, regardless of treatment group, displayed altered qPCR copy numbers compared to samples from a random dirty stall taken prior to cleaning. One farrowing stall was sampled per environment type from 4 different environmental surfaces (piglet heating pad, sow drinker, sow feeder, and sow floor), with each point representing an individual surface sample. (b and c) Postcleaning environmental samples from the nondisinfected stall (b) showed a tendency toward containing a high number of bacterial species as well as altered microbial compositions compared to the precleaning and disinfected environments (c). Each shape in panels a to c represents a single sample, while different shapes denote different surface types. (d) Bacterial diversities (Shannon’s *H*) among all sow sample types before farrowing were not significantly different between sows belonging to different farrowing environments. Differing superscripts denote differences (*P* < 0.05) in diversity among sample types. (e to j) Beta diversity analyses based on Bray-Curtis distances for sow vaginal (f), fecal (g), udder (h), oral (i), and milk (j) samples and all sow samples (e) combined revealed no significant compositional differences. Each point represents one individual sample, with shapes corresponding to treatment groups in panels d and f to j, while shapes in panel e correspond to sample types. Download FIG S1, TIF file, 0.2 MB. Copyright © 2021 Law et al. 2021 Law et al. https://creativecommons.org/licenses/by/4.0/ This content is distributed under the terms of the Creative Commons Attribution 4.0 International license.

### Farrowing stall disinfection influences gut and nasal microbiomes of piglets.

**FIG 2:**
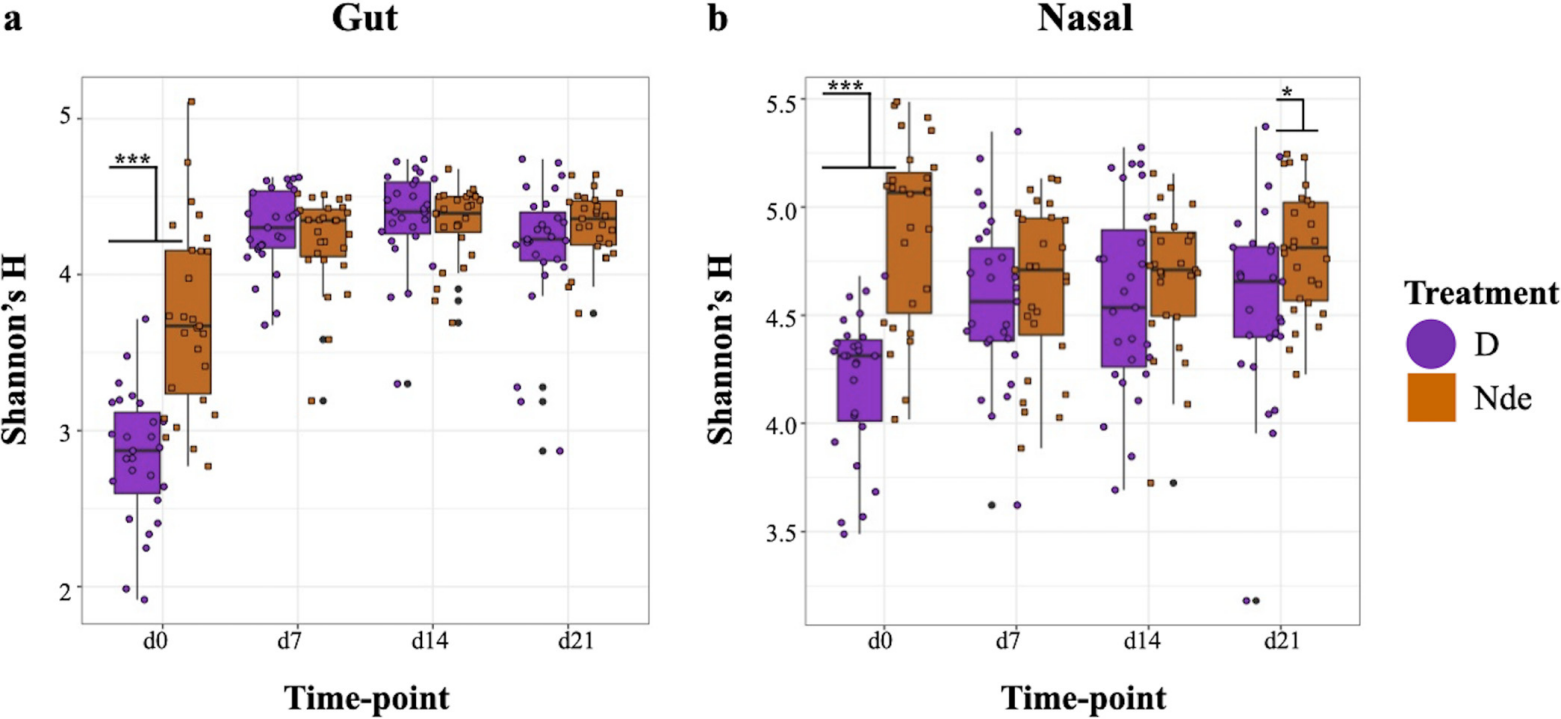
Bacterial diversity (Shannon’s *H*) between piglets born in the two different farrowing environments, assessed for piglet fecal (a) and nasal (b) samples from birth to weaning (day 0 to day 21).

Piglets born in environments devoid of disinfection (Nde) displayed differences in microbiome diversity and composition compared to those born in a disinfected farrowing environment (D). Bacterial diversity (Shannon’s *H*) in fecal samples was higher for piglets born in the nondisinfected environment at farrowing (day 0) (Shannon’s *H*, *P* < 0.001), although this effect was lost at subsequent time points (Fig. 2a). The same effect was replicated for nasal samples at day 0 (Shannon’s *H*, *P* < 0.001), with differences only observed again at day 21 (Shannon’s *H*, *P* < 0.05) (Fig. 2b).

**FIG 3:**
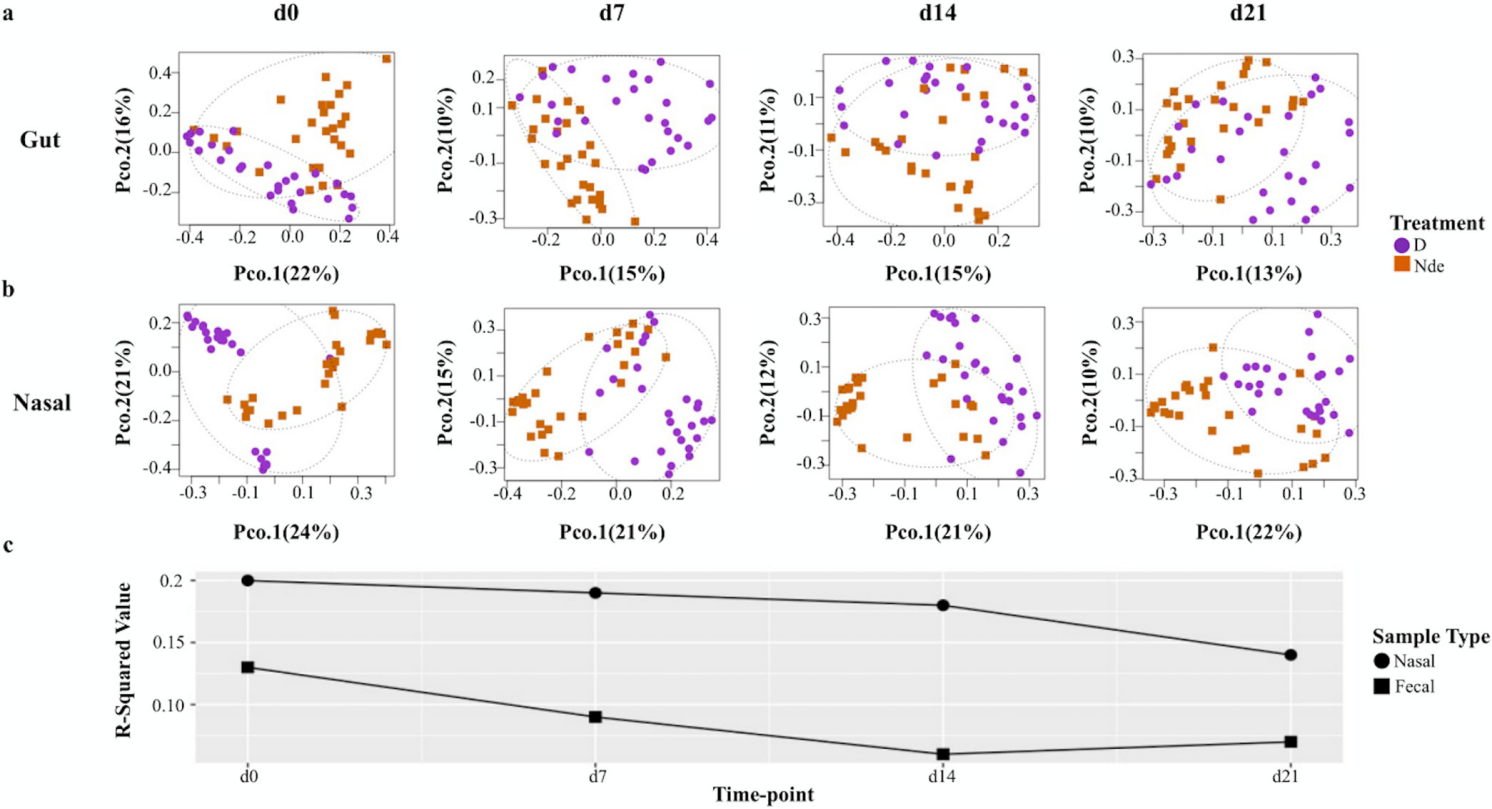
(a and b) Piglet gut (a) and nasal (b) microbiome compositions differed significantly from birth to weaning (Bray-Curtis, PCoA). Individual samples are represented by different shapes and colors according to farrowing stall sanitation. Dotted ellipses represent 95% confidence intervals in multivariate space. (c) Amount of variation of piglet microbiome composition explained by sanitation level in the farrowing environment (Bray-Curtis PERMANOVA, *R*^2^) for fecal and nasal samples.

Increased nasal and gut bacterial diversity in nondisinfected farrowing stalls at birth was also evident when splitting piglets by farrowing stall (litter), with the exception of stall 19 (Fig. S2a and b). Bray-Curtis distance ordination analysis of piglet fecal samples revealed clear compositional differences between piglets born in the two different farrowing environments (Fig. 3a) from birth to weaning (Bray-Curtis permutational multivariate analysis of variance [PERMANOVA], F-model = 4.3793 to 12.0072, *R*^2^ = 0.06 to 0.129, and *P *= 0.001).

10.1128/mSphere.00663-21.3 FIG S2 (a and b) Bacterial diversity (Shannon’s *H*) was assessed for individual stalls (litters) from birth to weaning (day 0 to day 21) for piglet fecal (a) and nasal (b) samples. Differing superscripts denote differences (*P* < 0.05) in diversity among individual stalls at that specific time point. (c and d) Influence of individual farrowing stall (litter) on piglet gut (c) and nasal (d) microbiome community compositions were assessed by grouping individual farrowing stalls (Bray-Curtis, PCoA) from birth to weaning (day 0 to day 21). Each point in panels c and d corresponds to an individual sample, with purple shades representing piglets born in the disinfected (D) farrowing environment and orange shades representing piglets born in the nondisinfected (Nde) farrowing environment. Download FIG S2, TIF file, 0.8 MB. Copyright © 2021 Law et al. 2021 Law et al. https://creativecommons.org/licenses/by/4.0/ This content is distributed under the terms of the Creative Commons Attribution 4.0 International license.

Analysis of piglet nasal samples revealed similar patterns (Fig. 3b), although the percentage of variation explained by disinfection was greater than that observed for fecal samples (Bray-Curtis PERMANOVA, F-model = 11.0959 to 33.157, *R*^2^ = 0.136 to 0.196, and *P* = 0.001). The *R*^2^ value decreased over time from birth to weaning for both the gut and nasal microbiomes (Fig. 3c). The consistently larger *R*^2^ values for nasal samples indicate a strong effect of farrowing stall disinfection on piglets’ nasal microbiomes compared with the same effect on gut microbial communities. Grouping of piglets by their respective farrowing stalls still shows a significant influence of farrowing stall disinfection on piglet nasal and gut microbiomes, although the individual stall shows a more significant effect size when used as an experimental block to create a new model (Bray-Curtis PERMANOVA, F-model = 3.6890 to 8.1779, *R*^2^ = 0.21791 to 0.3528, and *P* = 0.001 for the gut microbiome; Bray-Curtis PERMANOVA, F-model = 5.1663 to 21.953, *R*^2^ = 0.24677 to 0.51970, and *P* = 0.001 for the nasal microbiome) (Fig. S2c and d). However, just as observed with alpha diversity, disinfection did not have the same effects on beta diversity across all stalls, with piglets from stall/litter 19 (Nde) displaying distinct patterns compared to those from other nondisinfected stalls.

Beta diversity analyses also showed higher interindividual variation in the gut microbiome of piglets born in the disinfected (D) farrowing environment (Fig. S3) from birth to weaning at all time points (Wilcoxon test, day 0 *P* < 0.1, day 7 *P* < 0.01, day 14 *P* < 0.05, and day 21 *P* < 0.01). These observations were sustained only for piglet nasal microbiomes at days 7 and 14 (Wilcoxon test, day 7 *P* < 0.1 and day 14 *P* < 0.05).

10.1128/mSphere.00663-21.4 FIG S3 Interindividual variation between fecal (a) and nasal (b) samples from piglets born in each farrowing environment was calculated using Bray-Curtis beta diversity distances. Piglets born in the disinfected farrowing environment displayed a tendency toward greater interindividual variation. Download FIG S3, TIF file, 0.1 MB. Copyright © 2021 Law et al. 2021 Law et al. https://creativecommons.org/licenses/by/4.0/ This content is distributed under the terms of the Creative Commons Attribution 4.0 International license.

### Sanitation strategy in farrowing environments is associated with specific bacterial taxa.

Compositional differences in the microbiomes of piglets born in different farrowing environments were investigated further to identify taxa associated with specific sanitation environments. ASV-level sequence data were collapsed to generate genus-level taxa, and these taxa were used for species indicator analyses, as the top discriminant taxa at the ASV level consisted mainly of unidentified species. To be considered an indicator taxon, indicator values for each genus of >0.6 were deemed noteworthy, with values close to 1 displaying the best indicators. An indicator value of 1 indicates that the given genus is present in all samples of a group and occurs in high abundance compared to another group. The differential abundance of these taxa was validated with false discovery rate (FDR)-adjusted Wilcoxon rank sum tests (*q* < 0.05).

**FIG 4:**
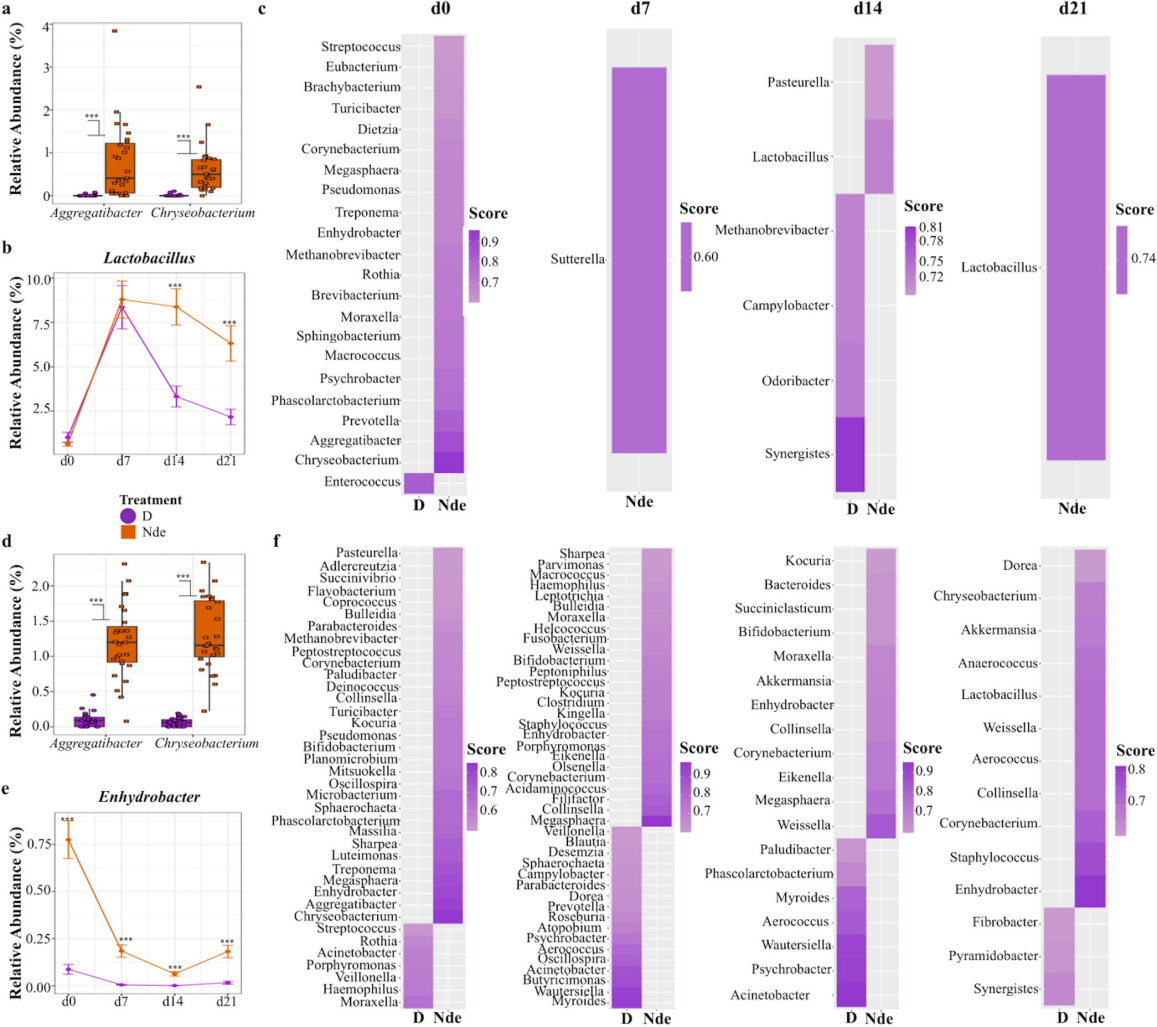
(a) Top discriminant genera at birth in piglet gut microbiomes between farrowing environments were observed to be two common environmental bacteria (*Aggregatibacter* and *Chryseobacterium*). (b) *Lactobacillus* was identified as a discriminant genus in piglet gut microbiomes at days 14 and 21. (c) Discriminant genera in piglet gut microbiomes with an indicator species score of >0.6 are displayed for all four time points. (d) The top discriminant genera at birth in piglet nasal microbiomes mirrored those present in piglet gut microbiomes. (e) The genus *Enhydrobacter* was identified as a discriminant genus in piglet nasal microbiomes at all four time points. (f) Discriminant genera for piglet nasal microbiomes with an indicator species score of >0.6 are displayed for all four time points. Darker shading in panels c and f correspond to higher indicator values.

The common environmental bacteria *Aggregatibacter* (20) and *Chryseobacterium* (21) were more abundant in the gut microbiomes of piglets born in the nondisinfected farrowing environment at birth (Wilcoxon test, *P* < 0.001) (Fig. 4a). About 7 days after birth (day 7), *Lactobacillus* increased sharply in abundance (>7-fold) for piglets born in both environments; however, compared to pigs born in disinfected farrowing stalls, piglets born in nondisinfected farrowing environments maintained a substantially higher abundance at days 14 and 21 (Wilcoxon test, *P* < 0.001) (Fig. 4b). Several other indicator genera for piglet gut microbiomes varied between farrowing environments. The abundances of native swine gut commensals such as *Prevotella* and *Phascolarctobacterium* (22) fluctuated in both groups across the 4 time points, while taxa with potential pathogenic or antibiotic resistance roles, such as *Enterococcus* (23) and Campylobacter (24), distinguished pigs born in the disinfected farrowing environment at specific time points (day 0 and day 14, for example, in Fig. 4c).

The top two discriminant genera in piglets’ nasal microbiomes at birth matched those found in gut microbiomes (Fig. 4d), with *Aggregatibacter* and *Chryseobacterium* also being significantly more abundant in piglets born in the nondisinfected farrowing environment. The genus *Enhydrobacter* was significantly more abundant in nasal microbiomes of piglets born in the nondisinfected farrowing environment throughout all four time points from birth to weaning (Fig. 4e). Other discriminant genera for piglet nasal microbiomes between farrowing environments for each time point are displayed in Fig. 4f. Among these, common swine nasal microbiome bacteria such as *Moraxella*, *Aerococcus*, and *Rothia* (25) fluctuated in abundance between piglets born in the different farrowing environments.

To further validate the discriminant genera identified by indicator species analysis, a RandomForest model was created using 500 trees. The out-of-bag (OOB) error rate for the models created based on piglet fecal samples fluctuated from 5.26% to 18.42% across the 4 time points. OOB error rates ranged from 0% to 5.25% for piglet nasal samples, also from day 0 to day 21. The model’s mean decrease Gini scores for the top 10 discriminant genera identified by indicator species analysis were recorded for each time point and sample type (Fig. S4a and b). Genera identified by the RandomForest model also mirrored discriminant genera identified by indicator species analysis, indicating that these taxa accurately classify piglets’ microbiomes according to the farrowing environment.

10.1128/mSphere.00663-21.5 FIG S4 Creation of a RandomForest model from genus-level relative abundances revealed minor differences in the identification of the top 10 discriminant genera between farrowing environments for both piglet fecal and nasal samples. The top 10 discriminant genera identified by indicator species analysis for fecal (a) and nasal (b) samples from birth to weaning (day 0 to day 21) are displayed and ranked according to the RandomForest model’s mean decrease Gini score and colored according to the treatment group in which they were more prevalent. Download FIG S4, TIF file, 0.2 MB. Copyright © 2021 Law et al. 2021 Law et al. https://creativecommons.org/licenses/by/4.0/ This content is distributed under the terms of the Creative Commons Attribution 4.0 International license.

### Network analyses reveal a strong association between farrowing environment and piglet microbiome community structure.

**FIG 5:**
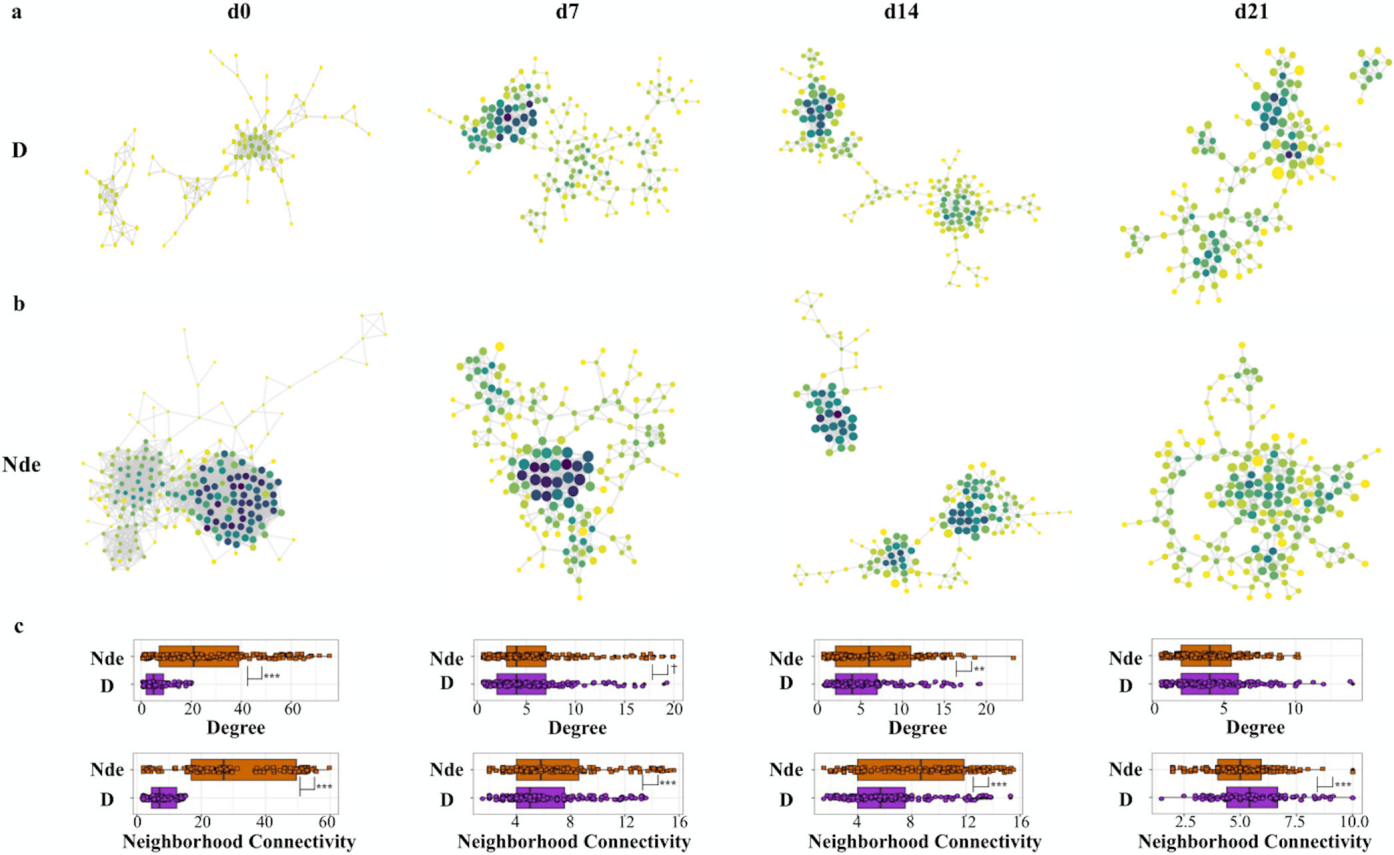
(a and b) Network analyses of piglet fecal samples from disinfected (a) and nondisinfected (b) farrowing environments. Each dot or node represents one taxon at the ASV level, with darker shading corresponding to higher degrees and larger node sizes corresponding to higher neighborhood connectivities. Edges represent the undirected interaction or correlation between two nodes or taxa. (c) Degree and neighborhood connectivity values were then quantified for each farrowing environment.

Networks that modeled microbiome community structure were created by using coabundance (correlation) matrices between microbes from each farrowing environment and were visualized in Cytoscape. Networks were curated to include only nodes or taxa with >10 correlations or edges. Coabundance network attributes for gut microbiomes differed significantly between piglets from the two farrowing environments (Fig. 5a and b and Fig. S5). Two network attributes with the most distinguishing patterns were degree and neighborhood connectivity (Fig. 5c), which measure the number of connections or associations that a node has and the average connectivity of all surrounding nodes in the network, respectively. Both of these network attributes were higher in gut microbiomes of piglets born in the nondisinfected farrowing environment (Nde), particularly at days 0 to 14 (Fig. 5c) (Wilcoxon test, day 0 *P* < 0.001, day 7 *P* < 0.1, and day 14 *P* < 0.01). The average shortest path length (ASPL), a measure of how fast information can travel through a network, was significantly higher in the gut microbiomes of piglets born in the disinfected farrowing environment from days 0 to 14 (Wilcoxon test, day 0 *P* < 0.01, day 7 *P* < 0.001, and day 14 *P* < 0.001) (Fig. S5). A higher ASPL, or a longer path length between nodes in a network, is associated with low relatedness between bacterial taxa and decreased functional redundancy, which may contribute to network and community instability (26). Nasal microbiomes of the piglets also showed different network attribute topologies, particularly with the observation of significantly higher ASPL values for piglets born in the disinfected farrowing environment at day 0 and day 7, mirroring what takes place at the gut level (Wilcoxon test, *P* < 0.001) (Fig. S6).

10.1128/mSphere.00663-21.6 FIG S5 Additional network attributes for network analyses created from taxa at the ASV level identified from piglet fecal samples, including the average shortest path length (ASPL) (a), closeness centrality (b), and eccentricity (c). Download FIG S5, TIF file, 0.6 MB. Copyright © 2021 Law et al. 2021 Law et al. https://creativecommons.org/licenses/by/4.0/ This content is distributed under the terms of the Creative Commons Attribution 4.0 International license.

10.1128/mSphere.00663-21.7 FIG S6 (a and b) Network analyses were created from taxa identified at the ASV level within piglet nasal samples from birth (day 0) to weaning (day 21) for piglets born in the disinfected (a) and nondisinfected (b) farrowing environments. Darker dot or node shading corresponds to a higher average shortest path length (ASPL). (c) Comparison of ASPLs of piglet nasal microbiome networks from each farrowing environment. Download FIG S6, TIF file, 0.9 MB. Copyright © 2021 Law et al. 2021 Law et al. https://creativecommons.org/licenses/by/4.0/ This content is distributed under the terms of the Creative Commons Attribution 4.0 International license.

All node network attributes were combined and analyzed using principal-component analysis (PCoA), displaying differences in all network attributes between ASVs in pigs born in disinfected and those born in nondisinfected farrowing stalls (Fig. S7) (Bray-Curtis PERMANOVA, F-model = 20.424 to 97.38, *R*^2^ = 0.06 to 0.22, and *P* = 0.001 for the gut microbiome; Bray-Curtis PERMANOVA, F-model = 15.528 to 68.507, *R*^2^ = 0.03 to 0.13, and *P* = 0.001 for the nasal microbiome), confirming that disinfection in the perinatal environment has significant effects on microbial community assembly and structure from birth to weaning.

10.1128/mSphere.00663-21.8 FIG S7 (a and c) Network attributes from piglet fecal (a) and nasal (c) networks were compiled and used to create PCoAs of each network at each time point based on Euclidean distances. Each shape corresponds to one node or ASV within the network. Dotted ellipses represent 95% confidence intervals for each group’s clustering pattern. (b and d) Box plots displaying PCoA scores along axis 1 for fecal (b) and nasal (d) network PCoAs. Download FIG S7, TIF file, 1.0 MB. Copyright © 2021 Law et al. 2021 Law et al. https://creativecommons.org/licenses/by/4.0/ This content is distributed under the terms of the Creative Commons Attribution 4.0 International license.

### Microbiome differences associated with the farrowing environment correspond to changes in growth performance.

**FIG 6:**
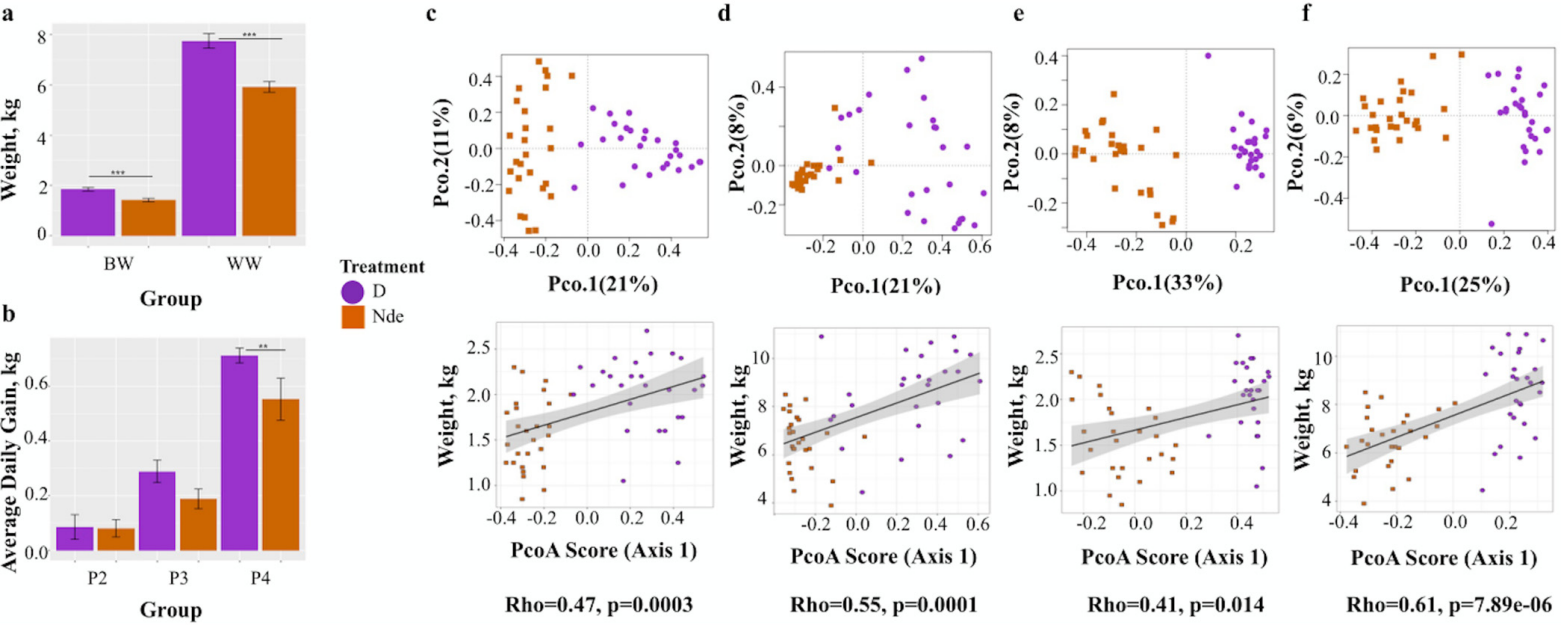
(a and b) Piglet growth performance from birth to weaning displayed through birth weights (BW) and weaning weights (WW) (a) as well as growth performance up to 6 weeks postweaning (b), with error bars representing standard errors. Period 1 (P1) represents the time period from birth to weaning (a), with P2 and P3 denoting weeks 1 and 2 postweaning, respectively, and P4 denoting the last 4 weeks of the nursery period. (c to f) PCoAs based upon discriminant ASVs between farrowing environments and Spearman correlations between piglet weights and PCoA scores along axis 1 at birth and weaning were created for piglet fecal (c and d) and nasal (e and f) samples, with shaded areas representing the 95% confidence intervals based on standard errors.

Piglets born in disinfected farrowing stalls displayed higher average weights at birth (1-tailed Student’s *t* test, *P* < 0.001) and weaning (1-tailed Student’s *t* test, *P* < 0.001) (Fig. 6a). The average daily gain (ADG) was also higher for these piglets after weaning, particularly for period 4 (1-tailed Student’s *t* test, *P* < 0.01) (Fig. 6b). These patterns were also observed when piglets were grouped by their individual farrowing stalls (Fig. S8a and b). Thus, the differences in growth performance between piglets from the two farrowing environments persisted through the nursery period.

10.1128/mSphere.00663-21.9 FIG S8 (a and b) Average piglet weights at birth and weaning (a) as well as piglet average daily gain (ADG) 1 week (P2), 2 weeks (P3), and 3 weeks (P4) after weaning (b) are displayed for each farrowing stall group, with error bars representing standard errors. (c to f) Spearman correlations were created between PCoA scores along axis 1 and piglet weights using the full set of ASVs at birth (day 0) and weaning (day 21) for piglet fecal (c and d) and nasal (e and f) samples, displaying less significant correlations than those created with the top discriminant ASVs. Download FIG S8, TIF file, 0.8 MB. Copyright © 2021 Law et al. 2021 Law et al. https://creativecommons.org/licenses/by/4.0/ This content is distributed under the terms of the Creative Commons Attribution 4.0 International license.

In light of the above-mentioned body weight and growth distinctions observed between piglets born in the different farrowing environments, we sought to trace possible associations between specific microbiome compositions and growth performances. To that end, we selected ASVs that best discriminated the gut and nasal microbiomes of piglets born in disinfected and nondisinfected environments at birth and weaning (indicator value of >0.6 and RandomForest mean decrease Gini value of >0.2) and used their abundances to generate a new set of PCoAs. As expected, these PCoAs yielded more pronounced discrimination between the microbiomes of piglets from both environments (Bray-Curtis PERMANOVA, F-model = 21.1, *R*^2^ = 0.29 to 0.54, and *P = *0.001) (Fig. 6c to f), providing further evidence that the selected discriminant taxa were major forces driving the separation along principal coordinate 1 (PCO.1). Next, Spearman correlations between the new ordination scores along PCO.1 and piglet weights at birth and weaning were measured, revealing significant associations between the abundances of discriminant taxa and physiological performance differences between piglets born in nondisinfected and disinfected farrowing environments (Rho = 0.41 to 0.61; *P* = 0.014 to 7.89e−06) (Fig. 6c to f). Significant associations between microbiome composition and piglet weight at birth and weaning were more pronounced when considering the top discriminant ASVs and not the full set (Rho = 0.13 to 0.37; *P* = 0.35 to 0.005) (Fig. S8c to f), indicating that the abundance of taxa associated with disinfection or the lack thereof in farrowing environments may be specifically associated with physiological performance.

## DISCUSSION

Here, we show that disinfection in the perinatal environment in swine production systems impacts the initial colonization of piglet microbiomes at birth, which could create lasting implications for gut and nasal bacterial microbiome compositions through weaning. Piglets born in a disinfected farrowing environment harbored distinctive genera in their gut and nasal microbiomes compared to those born in a farrowing environment cleaned only through power washing. Overall, piglet gut and nasal microbiome community structures and assemblies were significantly impacted from birth to weaning by disinfecting the perinatal environment, providing further evidence that changes in initial colonization may result in pervasive alterations in the development of infant microbiomes (27, 28).

### Farrowing stall sanitation affects environmental bacteria but has limited effects on sow microbiomes.

Disinfection of farrowing stalls before parturition is a common management technique to effectively reduce overall environmental bacterial loads and potential disease exposure risks for newborn piglets (29). The observed alterations in bacterial community composition, species richness, and bacterial 16S rRNA copy numbers (via qPCR) among environmental samples from farrowing stalls in different treatment groups postcleaning confirm that both sanitation and disinfection may have effectively predetermined microbial exposures for piglets at birth (see Fig. S1a to c in the supplemental material). However, the sows’ milk, gut, vaginal, nasal, oral, and udder skin microbiomes were not significantly affected by farrowing stall disinfection (Fig. S1d to j). This is contrary to previous findings comparing deliveries of infants in sanitized hospital and unsanitized home environments, characterized by distinct compositional differences in the vaginal microbiomes of the mothers (4). Because only 3 sows were included in each farrowing environment and the exposure to the D or Nde environment was relatively short for sows (∼3 days), neither the sample size nor the time of exposure may have been enough to identify microbiome differences in sow samples. However, the lack of differences detected in sow microbiomes could also be due to high interindividual variation in sows from disinfected environments (Fig. S1e to j).

### Farrowing stall disinfection persistently alters piglet gut and nasal microbiomes from birth to weaning.

Piglets born in the disinfected farrowing environment displayed decreased microbial diversity at birth (Fig. 2), further confirming that farrowing stall disinfection resulted in the alteration of microbial exposures. Although farrowing stalls were disinfected only prior to birth, compositional differences were observed between the gut and nasal microbiomes of piglets born in different farrowing environments from birth to weaning (Fig. 3a and b), indicating that disruption of the initial colonization process through environmental manipulations has lasting implications for piglet microbiome compositions. Previous studies regarding environmental manipulations in swine have also observed lasting effects on piglet microbiome composition, although these studies are limited in scope to the nursery period (15, 30). The first microorganisms responsible for the colonization of piglet gut microbiomes have previously been traced to several surfaces in the farrowing environment at birth (31), providing further evidence that environmental manipulations at birth have a direct impact on piglet microbiome composition. The observation that individual sow explained more compositional variation (Fig. S2c and d) than the farrowing environment supports previous reports that intrinsic factors related to individual sow are a significant determinant of piglet microbiome composition (32, 33), although the extent to which inherent maternal factors influence piglet microbiomes has yet to be determined. This issue is exemplified by the observation that alpha and beta diversity patterns for gut or nasal microbiomes of piglets born in stall 19 in the nondisinfected environment appear more similar to those displayed by piglets from disinfected stalls (Fig. S2), although stalls from the two environments were kept strictly separate (Fig. 1b).

Notably, piglets born in a nondisinfected farrowing environment displayed significantly higher abundances of the genus *Enhydrobacter* in their nasal microbiomes (Fig. 4e), which has been linked to inhibition of Salmonella persistence in microbial communities (34). Common commensal bacteria such as *Prevotella*, an important mediator of acetate production (35), were also significantly enriched in the Nde piglets’ gut microbiomes at birth (Fig. 4c). The enrichment of taxa such as *Prevotella* is notable because they are considered essential components of swine microbiomes due to their vital role in the fermentation and digestion of indigestible fibers that result in short-chain fatty acid (SCFA) production and the associated host health benefits (36). These benefits include lowering the pH of the gastrointestinal tract environment to help inhibit the growth of potential pathogens (competitive exclusion) and strengthening host immune defenses (36).

Piglets born in a nondisinfected farrowing environment also displayed significantly higher abundances of *Lactobacillus* in their gut microbiomes at days 14 and 21 than those born in a disinfected farrowing environment (Fig. 4b). *Lactobacillus* is a common commensal in gut microbiomes of swine that is purported to be an indicator of gut microbiome stability and health (37). Higher relative abundances of *Lactobacillus* have been associated with normal piglet birth weights (38) as well as improved ADG (39); however, these associations were not supported by our growth performance results (Fig. 6a and b and Fig. S8a and b). ADG is an important metric in pork production because it emphasizes the economic importance of keeping piglets healthy, as stressed piglets display a tendency toward decreased ADG and, therefore, decreased economic output (40). Higher relative abundances of *Lactobacillus* have also been associated with enhanced immune health and resilience in piglets, with observations of lower rates of postweaning diarrhea (38), increased resistance to Salmonella infection (41) and other enteric pathogens (42), and increased antioxidant capacity and immunoglobulin levels (43). Although higher abundances of *Lactobacillus* were not observed at birth in piglets born in the nondisinfected farrowing environment, the farrowing environment could have influenced a selective pressure for *Lactobacillus* to increase at day 14 and at weaning. Nonetheless, besides altered microbial exposures at birth, the current data cannot explain the reasons for the greater abundances of *Lactobacillus* only in the gut microbiomes of piglets born in nondisinfected farrowing stalls.

The increased abundance of *Lactobacillus* at weaning is interesting because of the physiological stress associated with weaning, where enhancing gut health and the immune system is critical (44). However, the reasons behind the enrichment of *Lactobacillus* in pigs born in nondisinfected environments warrant further exploration. Piglets born in the nondisinfected environment also harbored lower abundances of potentially pathogenic bacteria such as *Enterococcus* (23) and Campylobacter (24) at days 0 and 14 (Fig. 4c). These results are consistent with previous findings of greater abundances of species of *Lactobacillus* and reduced abundances of potentially pathogenic bacteria in piglets raised outdoors with more microbial exposures (15).

### Modulations in piglet microbiome compositions may have implications for long-term health and physiology.

Differences in the initial seeding of gut microbiomes of piglets born in disinfected and nondisinfected farrowing environments resulted in differential community assemblies and structures up to 21 days of age (Fig. 5 and Fig. S6), which could contribute to the observed differences in physiological performance. Piglet gut microbiomes undergo rapid colonization in the first 24 to 36 h after birth (45), and this critical developmental window can effectively predetermine lifelong microbiome compositions and health (46). Altered piglet gut microbiome compositions in early life have previously been associated with differences in growth performance (38, 39) and the metabolism of nutrients (30, 47, 48). Because gut tissues are energetically expensive to develop and maintain (49, 50), piglets born in the nondisinfected farrowing environment that were exposed to higher bacterial loads could have devoted more energy to developing immune competence or tolerating higher loads of colonizing microorganisms rather than allocating this energy for growth (51, 52). Additionally, the enrichment of genera associated with increased SCFA production in piglets born in the nondisinfected environment could be associated with energetically expensive upregulation of the host mucosal immune response (53), resulting in low energy being allocated to growth compared to piglets born in the disinfected environment.

The compositions of neonatal piglet microbiomes in early life are proposed to affect long-term piglet health and immunity (45). Susceptibility of piglets to postweaning diarrhea, an extremely damaging disease in swine production in the first few weeks after piglets’ transition to solid feed (54), has been associated with distinct compositional differences in piglet gut microbiomes as early as 7 days after birth (55). The piglet gut microbiome composition in the first few weeks of life has also been associated with the development of inflammatory responses in adulthood (46).

Disinfection practices in the farrowing environment resulted in microbiome disruption and differential colonization processes for piglets, as shown by cooccurrence network analyses (Fig. 5 and Fig. S6). Microbiome assembly and microbe-microbe interactions may have implications for immune function and susceptibility to disease later in life. Microbiome communities are complex networks of associations between many types of microorganisms occupying various ecological niches that remain relatively stable in composition after establishment (56). Microbiome stability is measured by the interrelatedness and functional capacity of the community, where increased associations and shared functions between taxonomic groups contribute to resilience to community perturbations (57, 58). Previous reports on networks modeling piglet gut microbiomes observed associations between growth performance and networks with strongly associated taxonomic clusters along with functional redundancy (59) as well as associations between microbiome community stability and SCFA production (60). Thus, higher taxon interrelatedness and stronger network associations in terms of high degree and network connectivity, low ASPL values, and less interindividual variability in both the gut and nasal microbiomes of piglets born in the nondisinfected farrowing environment indicate that these piglets’ microbiomes may be more stable and resilient to potential perturbations caused by future disease challenges. Piglets born in the disinfected farrowing environment displayed less microbial diversity at birth (Fig. 2), high interindividual variability, and low interrelatedness; therefore, they may be more sensitive to changing environmental conditions and community perturbations.

Our results indicate that disinfection of the farrowing environment and subsequent alteration of microbial exposures at birth may have the same implications for piglets as those proposed for humans in the hygiene hypothesis. The hygiene hypothesis (61) has long been used to explain associations between increased sanitation practices and increased incidences of allergic diseases in humans, with numerous reports confirming lower incidences of allergic diseases with higher perinatal (62) and childhood (63–65) microbial exposures. However, exposure and tolerance to a wide variety of microorganisms, not just those that cause disease, are necessary to adequately prime offspring microbiomes and immune systems (66, 67). Manipulation of this initial colonization process through decreasing microbial exposures in swine was previously associated with impaired gut microbiome establishment (28, 30, 68), increased proinflammatory responses (15, 16, 69), and decreased immunocompetence (70). Disinfection of the farrowing environment and the subsequent alteration of microbial exposures may result in the production of piglets with better growth in early life but lowered immunocompetence throughout their lives.

### Study limitations.

Although environmental samples collected before and after cleaning confirmed that disinfection effectively altered bacterial biomass and diversity in farrowing stalls, greater numbers of environmental and maternal samples from each specific farrowing stall of a larger number of sows are necessary to determine the forces responsible for seeding piglet microbiomes in the context of farrowing stall disinfection. Our results support the observation that disinfection alters the environmental microbiome; however, we cannot rule out the possibility that residual disinfectants in the environment may also have contributed to the observed differences in piglet microbiomes. Additionally, sows were housed in stalls postcleaning an average of only 3 days before farrowing, which could potentially diminish the effects of disinfection on the environmental microbial reservoir available to sows and piglets. Nonetheless, the effect of disinfection on piglet gut and nasal microbiomes was evident and was confirmed by the differential abundances of taxa such as *Lactobacillus* and *Enhydrobacter* through weaning. The high interindividual variation among piglets driven by litter membership also indicates that the inclusion of a larger sample size of sows and piglets is necessary to validate our results and capture accurate assessments of manipulation of farrowing environments on growth performance that account for intrinsic maternal factors and their effects on piglet microbiomes. Potential trade-offs between growth performance in early life and long-term immunological function and health must be evaluated and characterized through studies including immunological, health, and growth performance data that extend beyond weaning, before changes in standard management practices regarding farrowing stall disinfection are implemented.

### Conclusions.

Our results indicate that standard disinfection practices in the farrowing environment modulate the bacterial diversity and community composition of piglet gut and nasal microbiomes from birth to weaning. Data on environmental manipulations and early microbial exposures in swine production systems should guide standard management practices in the future, as the alteration of microbial exposures through different cleaning methods (71) may modify the microbiome in early life, with potentially lifelong productivity and health consequences. Future studies should include long-term characterization of piglet microbiomes and immunocompetence through disease challenges in disinfected and nondisinfected environments to further validate our results as well as the functional characterization of piglet microbiomes coupled with mechanistic studies to identify if and how piglet physiology is affected by the taxonomic changes presented here. Also, studies that evaluate the effects of different methods of sanitation in swine facilities, or microbially targeted therapeutics that help promote growth in nondisinfected environments, that reduce disease risk without compromising growth performance or microbial exposures in early life are warranted.

## MATERIALS AND METHODS

### Experimental design.

Landrace × Yorkshire crossbred sows (*n* = 6) were selected randomly and mated with Landrace or Duroc semen by artificial insemination. Sows were moved from group housing on straw bedding to the farrowing barn 1 week before farrowing, where they were individually housed in farrowing stalls on perforated flooring. Sows were equally separated into two different treatment groups based on farrowing stall disinfection levels prior to their entry into the stalls, one cleaned with hot-water power washing plus a common, broad-spectrum microbicidal commercial disinfectant (Virkon S; Lanxess) and one cleaned only with hot-water power washing (Fig. 1a). Each treatment group was housed in a separate room of the farrowing barn, with strict biosecurity practices between each room to prevent environmental contamination throughout the study (Fig. 1b). For disinfected stalls, the disinfectant was sprayed on all surfaces of the farrowing stalls after hot-water power washing and allowed to sit for 10 min in accordance with product instructions. Sow diets were formulated to meet or exceed nutrient recommendations set by the National Research Council (72) for gestating and lactating sows. Individual weights were recorded for piglets within 24 h of birth and at 3 weeks of age (weaning). At weaning, all piglets were allotted into pens (10 pigs/pen) within a confinement swine nursery and received the same industry-relevant diets formulated to meet or exceed nutrient recommendations set by the National Research Council (72) for nursery pigs. Individual pig weights and daily nursery pen feeder additions were also recorded for 6 weeks postweaning.

### Sample collection.

Sterile cotton swabs and collection tubes were used to collect all samples. Environmental sampling occurred before and after cleaning of the farrowing stalls. Swab samples were collected from one dirty farrowing stall (unsanitized) before cleaning as well as one farrowing stall per treatment group after sanitation through hot-water power washing either with or without disinfection. Four different surfaces were swabbed within each stall (Fig. 1a): farrowing stall floors, heating pad surfaces in piglet creep areas, sow drinkers, and sow feeders (*n* = 12). Environmental samples were collected using a standardized technique across all stall surfaces. Each surface was swabbed in a zigzag motion, making sure to cover the entire surface only once. Feeders, drinkers, and floors across all stalls were identical in terms of materials, dimensions, and specifications. Sow samples from vaginal, rectal, oral, and udder surfaces were collected at day 110 of gestation upon stall entry and the day before farrowing. Milk samples were collected from sows at day 7 postpartum (*n* = 6). Nine piglets were selected randomly from each sow for microbiome analyses (*n* = 54). Fecal and nasal swabs were collected from these piglets by the insertion of swabs just within the rectal or nasal cavities at day 0 within 24 h of birth, on days 7 and 14 postpartum, and on the day of weaning (day 21). All samples were immediately placed on dry ice after collection until they could be frozen at −80°C prior to DNA extraction.

### DNA extraction and sequencing.

DNA was extracted from samples using Qiagen PowerSoil DNA extraction kits. DNA extraction kits harbor their own distinct collection of microbes unique to each type of kit, subsequently dubbed the “kitome” (73). Potential contamination from DNA extraction kits and their accompanying laboratory tools complicates microbiome sequence analyses, necessitating the use of negative controls created with each set of reagents and a sterile blank cotton swab to characterize any potential reagent or environmental contamination. Sequence data were generated by targeting the V4 variable region of the 16S rRNA gene on the MiSeq sequencing platform using the primers 515F (5′-GTGCCAGCMGCCGCGGTAA-3′) and 806R (5′-GGACTACHVGGGTWTCTAAT-3′) and dual-indexing library preparation (74). Copy numbers of the 16S rRNA bacterial gene were quantified by qPCR using Kapa HiFi polymerase (Kapa Biosystems, Woburn, MA) with the following cycling conditions: 5 min at 95°C; 35 cycles of 20 s at 98°C, 15 s at 55°C, and 1 min at 72°C; and, finally, 5 min at 72°C. Custom-made Perl scripts (see Text S1 in the supplemental material) were used to process raw sequence data by removing primer sequences and quality filtering reads. Raw sequence data contained an average of 13,238 ± 2,945 forward/reverse reads per sample (range, 83 to 23,019 reads/sample), which was reduced to an average of 11,943 ± 3,184 reads per sample (range, 146 to 24,611 reads/sample) after processing and quality control procedures, with appropriate sequencing coverage across all samples (Data Set S1). Processed sequences were then run through the QIIME2 pipeline (75) and assigned amplicon sequence variants (ASVs) using the DADA2 plug-in (76) and the Greengenes database, v13_8 (77). The Greengenes database was selected for classification because of higher reported accuracy in regard to species- and genus-level annotations (78).

10.1128/mSphere.00663-21.1 TEXT S1 Custom Perl scripts used for sequence data processing. Scripts were used to locate and remove primer sequences and perform quality control on sequence data. Download Text S1, DOCX file, 0.01 MB. Copyright © 2021 Law et al. 2021 Law et al. https://creativecommons.org/licenses/by/4.0/ This content is distributed under the terms of the Creative Commons Attribution 4.0 International license.

10.1128/mSphere.00663-21.10 DATA SET S1 Sequencing summary statistics, including qPCR copy numbers, percentage of barcodes, percentage of one-mismatch batches, yield, quality, mean quality scores, and reads pre- and postprocessing. A rarefaction curve displays adequate sequencing depth and coverage for all samples, with the dotted line representing the average number of sample reads postprocessing. Each color represents one sample type. Download Data Set S1, XLSX file, 0.5 MB. Copyright © 2021 Law et al. 2021 Law et al. https://creativecommons.org/licenses/by/4.0/ This content is distributed under the terms of the Creative Commons Attribution 4.0 International license.

### Statistical analyses.

All statistical analyses comparing microbial compositions within each treatment group were performed using the R statistical interface (86). The negative controls created from DNA extraction kit reagents and sterile swabs were used to screen ASV-level sequence data for potential contamination using the R decontam package (87) based on the prevalence method, which identifies contaminants based on both the presence or absence of bacterial taxa in samples versus their corresponding control samples as well as the frequency at which they appear. ASVs identified as contaminants through this process were filtered out of sequence data sets. Sequence data were then filtered using the R labdsv package (78) to remove ASVs that were likely to be sequencing artifacts due to their presence at extremely low frequencies (*n* < 5) or in only 3 or fewer samples.

Alpha diversity analyses, beta diversity Bray-Curtis distances, PERMANOVA calculations, and calculations of interindividual variation were performed using the R vegan package (79). Principal-coordinate analyses based on Bray-Curtis or Euclidian distances as well as Spearman correlations among piglet weights and top discriminant taxa were created using the R ape package (80). Identification of discriminant taxa was performed using the R labdsv package (81), using a threshold of indicator values of >0.6. Indicator values represent the product of taxon mean abundances and their frequencies of occurrence, where an indicator value of 1 indicates that a given genus is present in all samples of a group and occurs in high mean abundances compared to another group (82). Discriminant taxa were also identified through RandomForest classification using 500 trees with the randomForest R package (83). The genera with the top 10 mean decrease Gini scores for each sample type at each time point were considered the top discriminant taxa, with a high mean decrease Gini score associated with the increased importance of a genus for the accurate classification of treatment groups within the model. Network analyses were performed by creating Spearman-based correlation matrices through the R package ccrepe (84), which were then loaded into the Cytoscape program (71). Individual box plots without jitter points and PCoAs were created using base R plotting functions, while all other figures were created using R ggplot2 (85). Statistical significance testing was performed using Wilcoxon tests, Kruskal-Wallis tests, or PERMANOVAs for all nonparametric microbiome-associated data and 1-tailed Student’s *t* tests for performance data or other parametric data. Statistical significance in all figures is denoted with three asterisks when the *P* value is <0.001, two asterisks when the *P* value is <0.01, one asterisk when the *P* value is <0.05, and a cross when the *P* value is <0.1.

### Data availability.

The 16S rRNA sequence data for this project were deposited in the NCBI Sequence Read Archive (SRA) under BioProject accession number PRJNA721243.
