# Supplementary material for: Disinfection of Maternal Environments Is Associated with Piglet Microbiome Composition from Birth to Weaning
Source: mSphere. 2021 Sep 8;6(5):e00663-21. doi: 10.1128/mSphere.00663-21 (PMC8550216; doi:10.1128/mSphere.00663-21)
Supplement: TEXT S1 [file msphere.00663-21-s0001.docx]

**Cutadapt processing to locate and remove primer sequences:**

for i in *_R1_001;

do

SAMPLE=$(echo ${i} | sed "s/_R1_\001\//")

echo ${SAMPLE}_R1_001 ${SAMPLE}_R2_001

cutadapt -a GTGCCAGCMGCCGCGGTAA...ATTAGAWACCCBDGTAGTCC -A GGACTACHVGGGTWTCTAAT...TTACCGCGGCKGCTGGCAC \

--discard-untrimmed -o /${SAMPLE}_R1_001 \

-p /${SAMPLE}_R2_001 ${SAMPLE}_R1_001 ${SAMPLE}_R2_001

done

**Remove empty lines created by Cutadapt:**

#!/usr/bin/perl

use strict;

use Data::Dumper;

my $hash={};

my $file = <$ARGV[0]>;

open (IF, $file);

open (OF, ">$file.Curated.fastq");

open (OF1, ">$file.EmptyLines");

while (chomp (my $line = <IF>))

{

#---- Always check M00262 should be in your annotation otherwise change it

if ($line =~ /M00262/)

{

#print $line; <stdin>;

chomp (my $line1 = <IF>);

chomp (my $line2 = <IF>);

chomp (my $line3 = <IF>);

if ($line1 =~ /A|G|C|T/)

{

print OF "$line\n$line1\n$line2\n$line3\n";

}

else

{

print OF1 "$line\n$line1\n$line2\n$line3\n";

}

}

}

**Quality filtering with fastx_toolkit:**

for i in *fastq; do fastq_quality_filter -q 30 -p 50 -i "$i" -o "$i".QC.gz -z; done;

**Repair disordered paired-end reads and formatting errors caused by QC processing:**

for i in *_R1_001;

do

SAMPLE=$(echo ${i} | sed "s/_R1_001//")

echo ${SAMPLE}_R1_001${SAMPLE}_R1_001

~/Softwares/bbmap/repair.sh

in1=${SAMPLE}_R1_001 \

in2=${SAMPLE}_R2_001 \

out1=${SAMPLE}_R1_001 \

out2=${SAMPLE}_R2_001 \

outsingle=${SAMPLE}_Singletons

done
